# The Versatile Type V CRISPR Effectors and Their Application Prospects

**DOI:** 10.3389/fcell.2020.622103

**Published:** 2021-02-04

**Authors:** Baisong Tong, Huina Dong, Yali Cui, Pingtao Jiang, Zhaoxia Jin, Dawei Zhang

**Affiliations:** ^1^School of Biological Engineering, Dalian Polytechnic University, Dalian, China; ^2^Tianjin Institute of Industrial Biotechnology, Chinese Academy of Sciences, Tianjin, China; ^3^Key Laboratory of Systems Microbial Biotechnology, Chinese Academy of Sciences, Tianjin, China; ^4^University of Chinese Academy of Sciences, Beijing, China

**Keywords:** CRISPR-Cas systems, type V effectors, genome editing, nucleic acid detection platforms, transcriptional regulation, base editor

## Abstract

The class II clustered regularly interspaced short palindromic repeats (CRISPR)–Cas systems, characterized by a single effector protein, can be further subdivided into types II, V, and VI. The application of the type II CRISPR effector protein Cas9 as a sequence-specific nuclease in gene editing has revolutionized this field. Similarly, Cas13 as the effector protein of type VI provides a convenient tool for RNA manipulation. Additionally, the type V CRISPR–Cas system is another valuable resource with many subtypes and diverse functions. In this review, we summarize all the subtypes of the type V family that have been identified so far. According to the functions currently displayed by the type V family, we attempt to introduce the functional principle, current application status, and development prospects in biotechnology for all major members.

## Introduction

The CRISPR (clustered regularly interspaced short palindromic repeats)–Cas (CRISPR-associated protein) system is an acquired immune mechanism, mostly found in bacteria and archaea as a defense against environmental mobile genetic elements (MGEs), such as phages, plasmids, and transposons (Sorek et al., 2013; Koonin et al., 2017). The CRISPR system has been divided into class I and class II according to the current classification method, and their effector nuclease modules are composed of multiple subunits or single proteins, respectively (Makarova et al., 2015). Class II is further divided into type II and type V with DNA interference activity, as well as type VI with RNA interference activity (Hajizadeh Dastjerdi et al., 2019).

Like other CRISPR–Cas systems, the type V system consists of three parts, i.e., the effector protein module, the acquisition module, and the CRISPR array (Sorek et al., 2013). When MGEs from the environment invade the host cell, Cas1 and Cas2 in the acquisition module form a complex to intercept a short segment of the invading genetic information named a protospacer, next to a short motif called the protospacer-adjacent motif (PAM), and insert it into a chain of repeated sequences in the CRISPR array (Yosef et al., 2012; Nunez et al., 2015). The CRISPR array is then transcribed to produce pre-CRISPR RNA (pre-crRNA), which is further processed by RNase III or effector proteins in its own CRISPR system to form mature CRISPR RNA (crRNA). Some CRISPR systems also transcribe a trans-activating crRNA (tracrRNA) that is mostly complementary to the repeat sequences in the CRISPR array. Mature crRNA is fused with tracrRNA, or crRNA alone acts as a guide RNA (gRNA) and combines with the effector nuclease in the effector module, which then matches the invading sequence and exerts targeted cleavage activity (Murugan et al., 2017).

The effector proteins of the type V family are diverse at the N-terminus but retain a unified RuvC-like endonuclease (RuvC) domain at the C-terminus, derived from the TnpB protein encoded by autonomous or non-autonomous transposons (Shmakov et al., 2017). According to the dissimilar CRISPR effectors, the type V system is further subdivided into many subtypes, including types V-A to V-I, type V-K, type V-U, and CRISPR–CasΦ (Hajizadeh Dastjerdi et al., 2019; Pausch et al., 2020). In these subtype systems, the corresponding effector proteins have shown a variety of functions, whereby some can act not only on double-stranded DNA (dsDNA) but also on single-stranded DNA (ssDNA) and single-stranded RNA (ssRNA). This multifunctionality has put the type V CRISPR–Cas system into the focus of recent studies. However, there is still no review that systematically summarizes these various type V CRISPR systems. The overall sorting is conducive to the application of type V systems and a profound understanding of CRISPR systems, which we wish to promote with this review.

## History of The Type V Family

Type V-A, which was identified as the first known type V system in the human pathogenic bacterium *Francisella tularensis* in 2013, has the signature effector protein Cas12a (formerly designated Cpf1) (Schunder et al., 2013). Directed by gRNA, Cas12a successively cleaves the non-complementary and complementary strands of the targeted DNA segment using a single RuvC nuclease domain (Jeon et al., 2018). This is significantly different from the type II CRISPR enzyme Cas9, which uses the HNH-nuclease domain and RuvC-like nuclease domain to cut the two strands of the targeted DNA segment, respectively (Sternberg et al., 2015). The C-terminus with only one RuvC endonuclease domain is the defining characteristic of the type V CRISPR system (Makarova et al., 2015).

In order to explore more unknown type V systems, researchers designed a sophisticated *in silico* analysis approach using the Cas1 protein, which is widespread and highly conserved among the CRISPR–Cas systems, as the “seed” to query the sequence information in the available terabase-scale database. Through this method, Shmakov et al. identified type V-B and type V-C in 2015 (Shmakov et al., 2015), Harrington et al. identified type V-F in 2018 (Harrington et al., 2018), and Yan et al. identified type V-G, type V-H, and type V-I in 2019 (Yan et al., 2019). By querying uncultivated bacteria in groundwater and sediment samples *via* genome-resolved metagenomics, Burstein and colleagues identified type V-D and type V-E in 2017 (Burstein et al., 2017).

The other way to identify CRISPR–Cas systems, including type V-U systems and the CRISPR–CasΦ system, is using CRISPR arrays with a fixed structural pattern as the “anchor.” There is no acquisition module near these CRISPR locus, which indicates that these systems cannot be identified by Cas1 and also indicates that there is a novel mechanism different from Cas1 and Cas2 in the adaptation phase of these CRISPR systems. Shmakov et al. discovered a series of novel type V-U systems, including V-U1, V-U2, V-U3, V-U4, and V-U5 (Shmakov et al., 2017). The spacer in the CRISPR array is consistent with some phage motifs, providing evidences for the activity of this CRISPR locus. In another investigation, researchers were surprised to find that the type V-U5 system is closely associated to a Tn7-like transposon (Faure et al., 2019). This unusual characteristic makes type V-U5 different from type V-U, and it was reclassified as the independent subtype V-K. In huge phages existing in diverse ecosystems, Al-Shayeb et al. identified type II, type V-I, type V-U, and type V-F, which are never found in phages, and the CRISPR–CasΦ system, which is not reported in bacteria and archaea. In these systems, their spacers target the host chromosome or other competitive phages, which facilitate phage invasion (Al-Shayeb et al., 2020).

**Figure 1:**
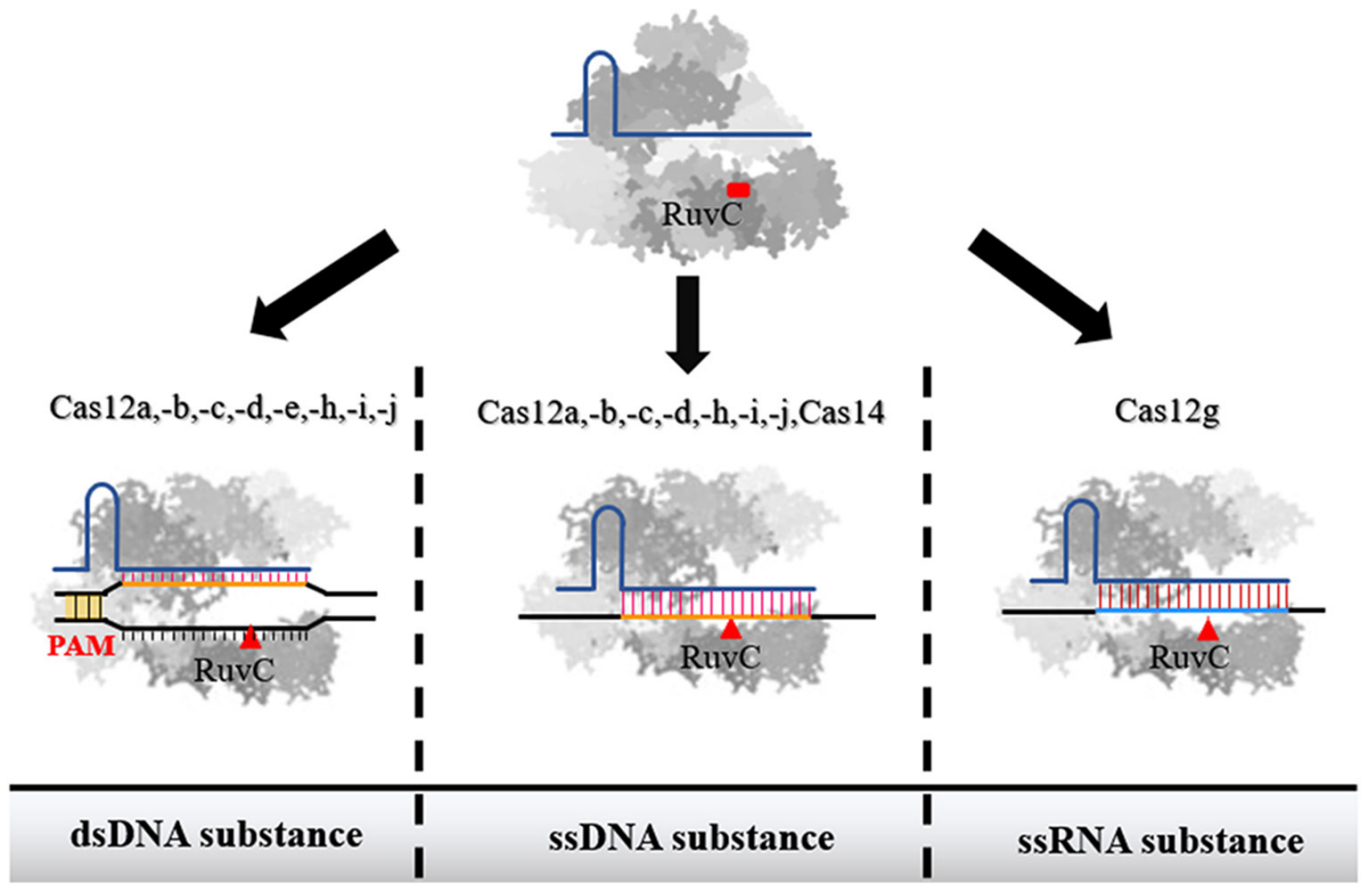
The recognition substance of effector proteins of each subtype in type V family. Among them, Cas12a, Cas12b, Cas12c, Cas12d, Cas12h, Cas12i, and Cas12j were confirmed to have the ability to interact with both dsDNA and ssDNA. Cas14 only has the ability to act on ssDNA. Cas12g can recognize ssRNA. When the single-stranded nucleic acid molecule is used as a substance, the binding of Cas protein is not restricted by PAM.

As described in the Introduction section, these different type V subtypes can interact with three different substrate types (dsDNA, ssDNA, and ssRNA). In addition, different subtypes also exhibit different characteristics with the same substrate (Table 1). In the following section, we classify the subtypes according to different target substrates and discuss the application potential of various type V subtypes (Figure 1).

**Table 1:** Properties of type V family effectors.

**Subtype**	**Signature protein**	**Length (aa)**	**PAM**	**tracrRNA/scoutRNA**	**Processing Pre-crRNA**	**Target substrates**	**Trans-cleavage**	**References**
Type V-A	Cas12a	1,200–1,500	(T)TTV^a^	No	Yes	dsDNA and ssDNA	ssDNA	Zetsche et al., 2015
Type V-B	Cas12b	~1,300	TTN	tracrRNA	No	dsDNA and ssDNA	ssDNA	Shmakov et al., 2015
Type V-C	Cas12c	1,200–1,300	TG or TN	scoutRNA	Yes	dsDNA and ssDNA	ssDNA	Yan et al., 2019; Harrington et al., 2020
Type V-D	Cas12d	~1,200	TA or TG	scoutRNA	Unconfirmed	dsDNA and ssDNA	ssDNA	Chen L. X. et al., 2019; Harrington et al., 2020
Type V-E	Cas12e	~1,000	TTCN	tracrRNA	Unconfirmed	dsDNA	ssDNA	Liu J. J. et al., 2019
Type V-F	Cas14	400–700	NO	tracrRNA	No	ssDNA	ssDNA	Harrington et al., 2018
Type V-G	Cas12g	~800	NO	tracrRNA	No	ssRNA	ssRNA and ssDNA	Yan et al., 2019
Type V-H	Cas12h	~900	RTR^b^	No	No	dsDNA and ssDNA	ssDNA	Yan et al., 2019
Type V-I	Cas12i	~1,100	TTN	No	Yes	dsDNA and ssDNA	ssDNA	Yan et al., 2019
Type V-K	Cas12k	~650	GTN	tracrRNA	No	dsDNA	No	Strecker et al., 2019b
CRISPR–CasΦ	Cas12j	~750	TBN^c^	No	Yes	dsDNA and ssDNA	ssDNA	Pausch et al., 2020

a *V represents A, C, and G*.

b *R represents A and G*.

c *B represents C, G, and T*.

## Targeting dsDNA by The Type V Family

To date, most of the CRISPR systems identified as belonging to the type V family have demonstrated targeted RNA-guided dsDNA cleavage activity. Cas12a (type V-A), Cas12b (type V-B), and Cas12e (type V-E) have been studied in detail, and their structural and functional characteristics are relatively clear. After the effector protein binds the gRNA to form a binary complex, it specifically recognizes the 5′ T-rich PAM and promotes target DNA unwinding, specified by Watson–Crick base pairing with the guide sequence of crRNA (Stella et al., 2017). At the same time, the non-target strand (NTS) of the target sequence is displaced, forming a so-called “R-loop” structure. The RuvC domain cuts the NTS and target strand (TS) successively at PAM-distant sites to form a staggered incision with 5, 7, or 10 nt 5′ overhangs (Yang et al., 2016; Liu J. J. et al., 2019). Notably, this is different from Cas9, which acts on the PAM-proximal bond to produce a blunt end (Jinek et al., 2014). Cas12c, Cas12d, Cas12h, Cas12i, Cas12k, and Cas12Φ also belong to the type V family but are less known. In addition, some of these type V Cas proteins have dual-nuclease activity, such as Cas12a, Cas12c (Harrington et al., 2020), Cas12i (Yan et al., 2019), and Cas12j (Pausch et al., 2020). They can process pre-crRNA by themselves, which makes them uniquely suitable for multichannel manipulation (Fonfara et al., 2016; Zetsche et al., 2017; Zhang et al., 2017). Although Cas9 can also modify multiple genes at the same time, either multiple separate sgRNA units (including promoter, gRNA, and terminator) or complicated modifications to the CRISPR array are needed, which makes the process cumbersome and inconvenient (Nissim et al., 2014; Sakuma et al., 2014).

### Cas12a, Cas12b, and Cas12e Domain Organization and Divergence

**Figure 2:**
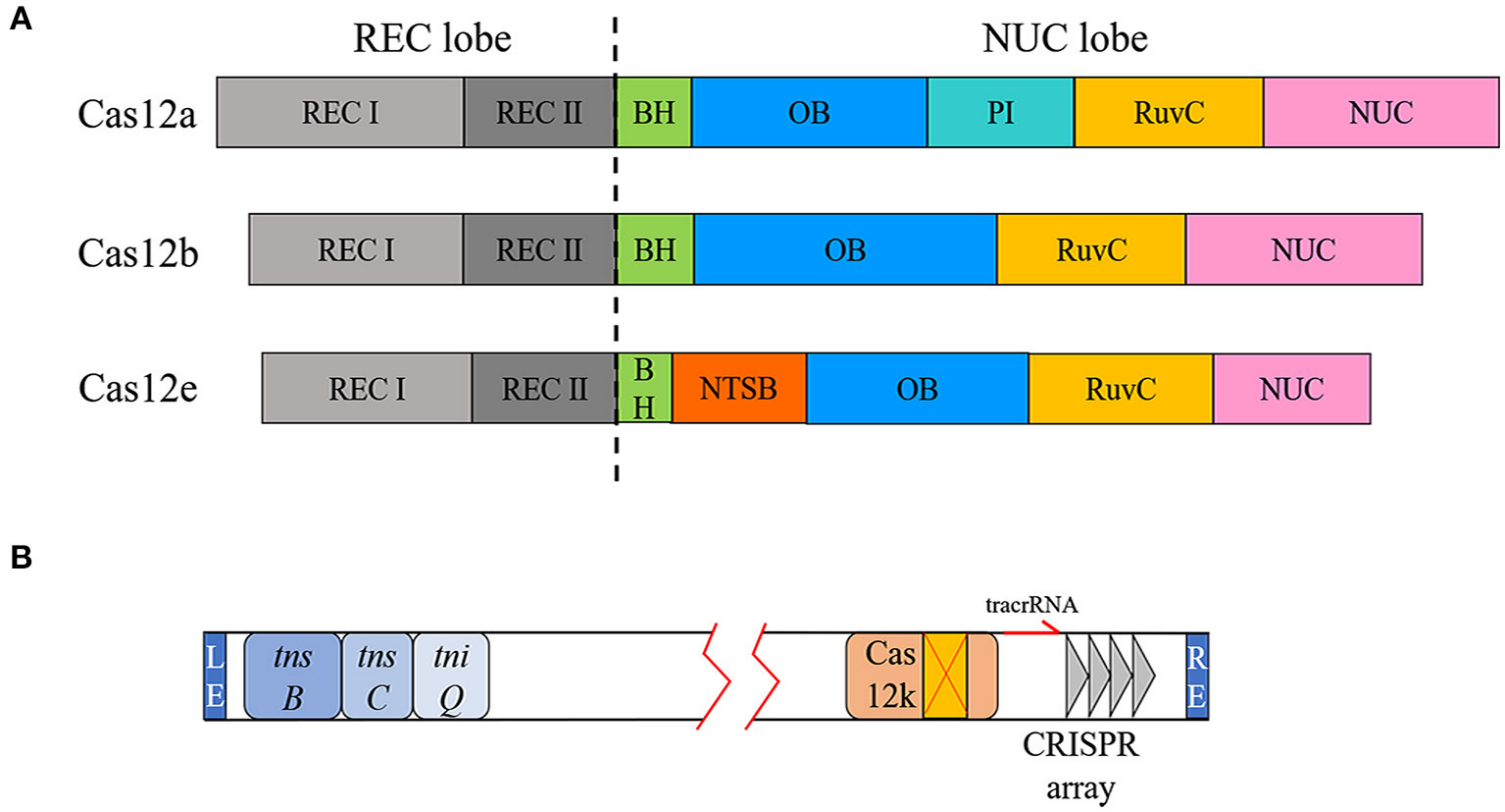
Domain diversity of Cas12a, Cas12b, and Cas12e and type V-K CRISPR loci. **(A)** The REC lobes of Cas12a, Cas12b, and Cas12e all contain two REC domains. There are some differences in the NUC lobe, including the presence of the PI domain and the additional NTSB domain of Cas12e. **(B)** Transposon-related genes and CRISPR elements are located at both ends of the transposon.

All three proteins have bilobed structures composed of an α-helical recognition (REC) lobe and a nuclease (NUC) lobe. The two lobes are connected by a bridge helix (BH) motif (Figure 2A). The REC lobe contains two REC domains (REC1 and REC2), which mainly help accommodate and stabilize the crRNA-target DNA hybrid after forming the “R-loop” (Gao et al., 2016). In the NUC lobe, Cas12a contains a RuvC domain, a PAM identical (PI) domain, an oligonucleotide-binding (OB) domain, and a NUC domain. The NUC domain was previously considered to be another nuclease domain of type V family enzymes, but recently, researchers have proposed to rename it as the TSL (target strand loading) domain to better reflect its actual role (Liu J. J. et al., 2019). The OB domain of Cas12a combines with the secondary structure at the 5′ end of gRNA to form a binary complex, and the initial 5 nt of crRNA in the PAM-proximal have an A-form-like conformation (Dong et al., 2016; Swarts and Jinek, 2019). These several pre-ordered nucleotides are defined as the “seed sequence.” When the PI domain recognizes the PAM and unzips the targeted dsDNA, the seed sequence immediately interact with the target strands, laying a foundation for the extension of the R-loop (Stella et al., 2017). Consequently, mismatches in the seed sequence region are often devastating for the targeting function of the Cas protein. Cas12b does not have an independent PI domain. After combining with gRNA to form a binary complex, the OB domain and REC-I will form a pre-organized PAM duplex cleft, where helices α5 and α6 and the flexible loop between α4 and α5 in the REC-I occupy a similar position to that of the PI domain in Cas12a and identify PAM (Yang et al., 2016; Wu et al., 2017). Cas12e also contains the unique non-target strand binding (NTSB) domain, which is required for target DNA unwinding. This additional domain suggests that the Cas12e–gRNA complex recognizes the target sequence and combines with it to form a ternary complex *via* a mechanism different from those of other CRISPR enzymes (Liu J. J. et al., 2019).

### Other Cas Proteins That Recognize dsDNA

Some newly discovered Cas proteins from the type V family also have dsDNA cleavage activity, albeit only proven by biochemical experiment, including Cas12c, Cas12d, Cas12h, Cas12i, and Cas12j (Chen L. X. et al., 2019; Yan et al., 2019; Pausch et al., 2020). It is worth noting that although Cas12i induces double-strand breaks (DSB), the efficiency of cutting is different for each strand of the target DNA, with strong cleavage of the non-complementary strands, but very low cleavage efficiency for complementary strands. Consequently, DSB are only produced at very limited frequencies (Yan et al., 2019). This feature makes it possible to use Cas12i as a nickase by using a pair of gRNAs to reduce the off-target rate and achieve large-scale gene fragment manipulation just like nCas9 (Ran et al., 2013a; Standage-Beier et al., 2015).

In addition to targeting dsDNA for cleavage, the type V-K system can target dsDNA for transposition. As mentioned above, the type V-K CRISPR locus is associated with Tn7-like transposons (Faure et al., 2019). The range framed by the transposon end sequences (LE and RE) encodes a Cas12k enzyme with a naturally inactivated RuvC domain and the three transposition-related genes *tnsB, tnsC*, and *tniQ* (Figure 2B). Previous researches indicated that canonical Tn7 transposons integrate the cargo gene between LE and RE at a specific Tn7 attachment site (*attTn7*) at higher frequencies or randomly integrate through another mutually exclusive mechanism (Peters, 2014). However, the insertion site of the transposon-encoded CRISPR system is located at a fixed distance downstream of the PAM of the targeting locus. Cas12k, which has lost its DNase activity, does not simply play an RNA-directed recognition role. When Cas12k was replaced with Cas9, there was no transposition phenomenon, indicating that Cas12k and the transposase complex have a coupling activation effect (Strecker et al., 2019b). Although Cas12k does not destroy the target site after binding to the target DNA, it has the same targeted immunity effect as the canonical Tn7 transposase in preventing multiple insertions. Consequently, the type V-K system will only insert the cargo gene once in a certain orientation at the same position (Stellwagen and Craig, 1997; Skelding et al., 2003).

### Applications Based on dsDNA Recognition

#### Genome Editing

**Figure 3:**
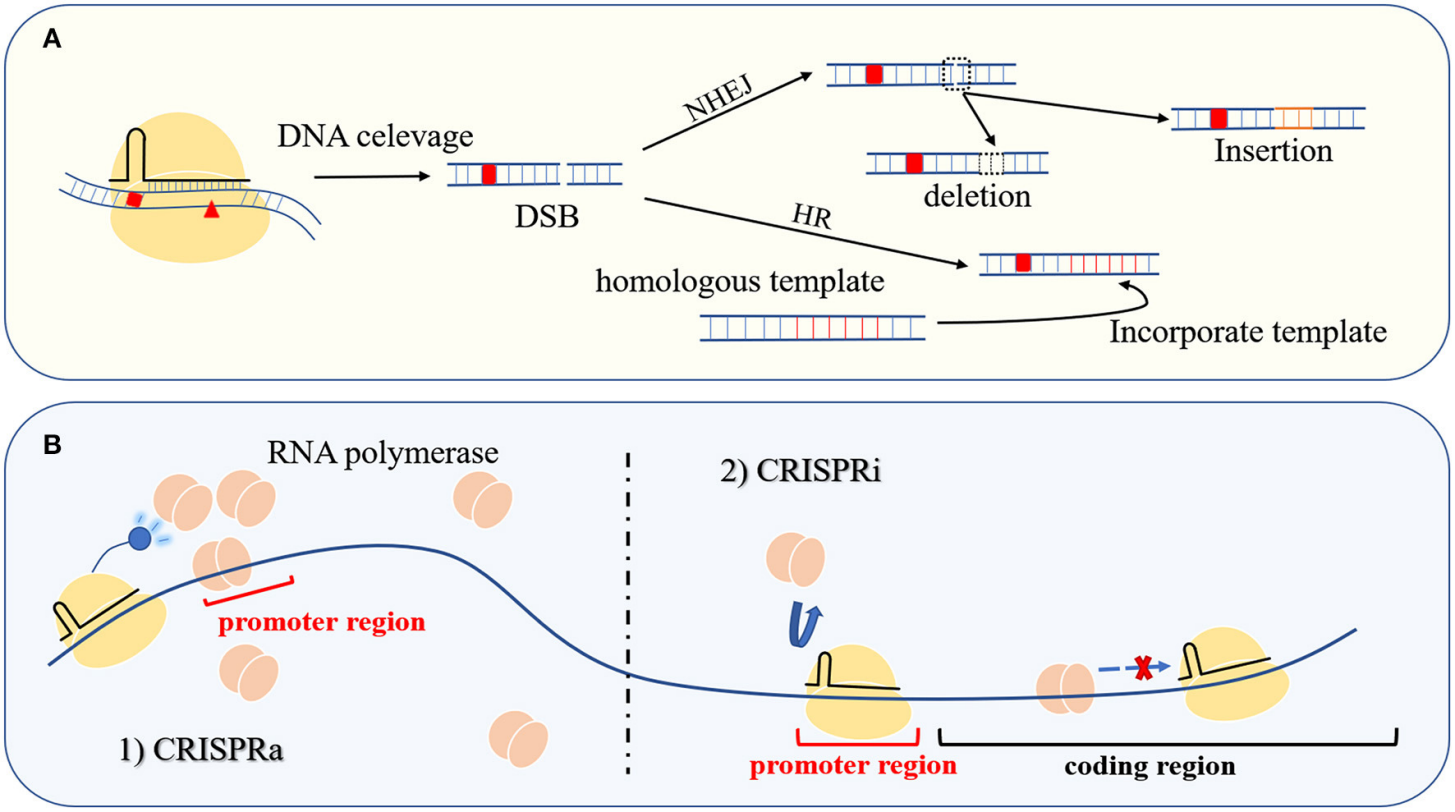
Gene editing and transcriptional regulation. **(A)** The Cas protein at the user-specified site causes DSB to achieve seamless operation at the targeted location through NHEJ or HDR. **(B)** The left side of the picture is CRISPR-mediated transcription activation (CRISPRa), and the right side is CRISPR-mediated transcription repression (CRISPRi). Control gene transcription is through gain and loss of RNA polymerase, respectively.

As an RNA-dependent sequence-specific nuclease, the Cas protein was developed as a gene editing tool at the beginning of research in this field (Ran et al., 2013b). Single or multiple plasmids express effector protein and artificial gRNA, induce DSB in a user-specified manner, and repair the DNA damage through non-homologous end joining (NHEJ) or homology-directed recombination (HDR) (Pawelczak et al., 2018). In the absence of a DNA template, the imprecise NHEJ-based repair will introduce random indels of several nucleotides at the cleavage site. The resulting frameshift mutations usually lead to gene inactivation. In the presence of homologous templates, HDR can achieve gene replacement, insertion, deletion, and other traceless genetic manipulations (Figure 3A). To date, the genetic manipulation toolbox based on Cas12a and Cas12b from the type V family has been successfully applied in mammals, plants, and bacteria (Table 2).

**Table 2:** Summary of organisms whose genomes were successfully edited using Cas12a and Cas12b.

**Bacteria**		**Animal**	**Plant**
• *Saccharomyces cerevisiae* (Li Z. H. et al., 2018) • *Yarrowia lipolytica* (Yang et al., 2020) • *Ashbya gossypii* (Jiménez et al., 2020) • *Aspergillus nidulans* (Vanegas et al., 2019) • *Clostridium beijerinckii* (Zhang J. et al., 2018) • *Clostridium difficile* (Hong et al., 2018) • *Corynebacterium glutamicum* (Zhang et al., 2019)	• *Zymomonas mobilis* (Shen et al., 2019) • *Pseudomonas putida* (Sun J. et al., 2018) • *Streptomyces coelicolor* (Li L. et al., 2018) • *Mycobacterium smegmatis* (Sun B. et al., 2018) • *Bacillus subtilis* (Wu Y. et al., 2020) • Cyanobacterium *Anabaena* (Niu et al., 2019) • *Chlamydomonas reinhardtii* (Ferenczi et al., 2017)	• Human cell (Strecker et al., 2019a; Teng et al., 2019b) • Zebrafish (Fernandez et al., 2018) • Mice (Kim Y. et al., 2016; Teng et al., 2018) • Pig (Zou et al., 2019) • *Drosophila* (Port and Bullock, 2016)	• Cotton (Li et al., 2019) • Rice (Yin et al., 2019; Ming et al., 2020) • Tobacco (Endo and Toki, 2019) • Maize (Malzahn et al., 2019) • Tomato (Vu et al., 2020) • Citrus (Jia et al., 2019) • *Arabidopsis thaliana* (Wu F. et al., 2020)

They, as supplements to Cas9, have been applied in many organisms that are not compatible with Cas9 (Ungerer and Pakrasi, 2016; Jiang et al., 2017). The Cas proteins mentioned above that have only been verified by biochemistry with dsDNA-targeted cleavage capabilities also have the ability to act as alternatives. These type V Cas proteins recognize a completely different PAM from Cas9, extending the targeting range, and the sticky ends generated at the PAM-distal position contribute to the efficiency of HDR repair (Fagerlund et al., 2015; Moreno-Mateos et al., 2017). Furthermore, compared with Cas9, Cas12a shows a lower frequency of off-target events (Kim D. et al., 2016; McMahon and Rahdar, 2021).

The CRISPR–Cas system is more convenient to operate in gene editing compared with the previous two generations of targeted gene editing technology, zinc-finger nucleases (ZFN), and transcription activator-like effector nucleases (TALENs), but it is not without its own limitations. The DSB induced by the sequence-specific nucleases at the target sites are fatal in organisms without effective NHEJ or HDR (Xu et al., 2015). To overcome this limitation, researchers attempted to connect different transposases to catalytically inactivated dCas9 (site-specific mutations were introduced into the HNH and RuvC domains to abrogate the cleavage activity without affecting the targeted binding effect) to obtain a series of dCas9–transposase fusion proteins [such as dCas9-*Himar1* (Chen and Wang, 2019), dCas9-*piggyBac* (Hew et al., 2019), and dCas9-*Sleeping Beauty* (Kovač et al., 2020)]. These fusions limit the random insertion of transposase, so that transposition occurs only near the point where dCas9 is targeted. Although the transposition reaction does not depend on the DSB repair ability of the host, limitations of the fusion protein by the PAM of dCas9 and the transposase insertion hot spot make this tool less flexible. For example, dCas9-Himar1 requires a TA dinucleotide within 15 bp behind the 5′ end of the gRNA (Chen and Wang, 2019).

The insertion site of the type V-K CRISPR system is only related to the target position of the gRNA, and there is no hot spot preference (Strecker et al., 2019b). This makes the operable range of this system more extensive, and the type V-K system has already been successfully used as a programmable gene editing tool in *Escherichia coli*. Through biochemical experiments, it was found that the Cas12k enzyme of this transposon-associated type V CRISPR system preferentially recognizes the 5′-GTN PAM. Cargo genes are inserted unidirectionally at positions 60–66 downstream of the PAM, and the insertion efficiency of a 2-kb cargo gene can be up to 80% without antibiotic screening. Although the type V-K system that can integrate exogenous genes into a certain position relies on a self-sufficient mechanism, both the LE/RE and the 5-bp repeat sequence generated by transposition are inevitably inserted together, which leads to redundancy in the genome (Strecker et al., 2019b). This is a major obstacle to the widespread application of type V-K and the focus of future optimization.

#### Transcriptional Regulation

Mutation of active site residues in the RuvC nuclease domain of the Cas nucleases in the type V family can be used to produce deactivated Cas proteins (dCas). Guided by gRNA, the dCas protein only binds to the target site and does not exhibit nuclease activity. When dCas targets the promoter region or coding region of a structural gene, it inhibits gene transcription by competing with RNA polymerase for promoter binding or by blocking polymerase migration. The resulting gene repression method is known as CRISPR interference (CRISPRi) (Figure 3B) (Kim et al., 2017; Zhang et al., 2017). In addition, dCas proteins can be used as targeting moieties for delivering a functional protein to a specific loci, including transcription factors, transcriptional activators, and RNA polymerase subunits. When the fusion protein is targeted to the proper distance from the gene transcription start site (TSS), the gene expression can be improved through the interaction between these functional proteins and RNA polymerase. This transcriptional activation tool based on the CRISPR–Cas system is called CRISPRa (Figure 3B). The emergence of CRISPRi and CRISPRa provides convenient tools for the characterization of gene functions and metabolic regulation (Liu W. et al., 2019; Martella et al., 2019).

As mentioned above, the RNase activity of Cas12a can process a customized CRISPR array. Zhang et al. constructed the CRISPR array to target four locus, and dCas12a successfully inhibited multiple genes simultaneously. Moreover, the efficiency of simultaneously repressing four genes was similar to that of repressing the separated gene (Zhang et al., 2017). Zhang J. L. et al. (2018) used three crRNAs to coordinately target the same gene, which increased the repression efficiency by 6.7-fold. In addition, Cas12a has been developed into multipurposed tools. For example, Adithya et al. found that when the crRNA length is <16 bp, Cas12a can bind to the target site without cleavage. On this basis, the gene editing and CRISPRi can be realized simultaneously by transferring crRNAs with different lengths in series (Ramesh et al., 2020). Wu et al. achieved repression and activation on multiple genes simultaneously. When dCas12a-RemA (a kind of transcription factor) bound to the crRNA targeted into genes, it successfully downregulated the genes. When dCas12a-RemA bound to the crRNA targeted upstream of the TSS, it successfully upregulated the expression of the gene (Wu Y. et al., 2020).

#### Base Editing

**Figure 4:**
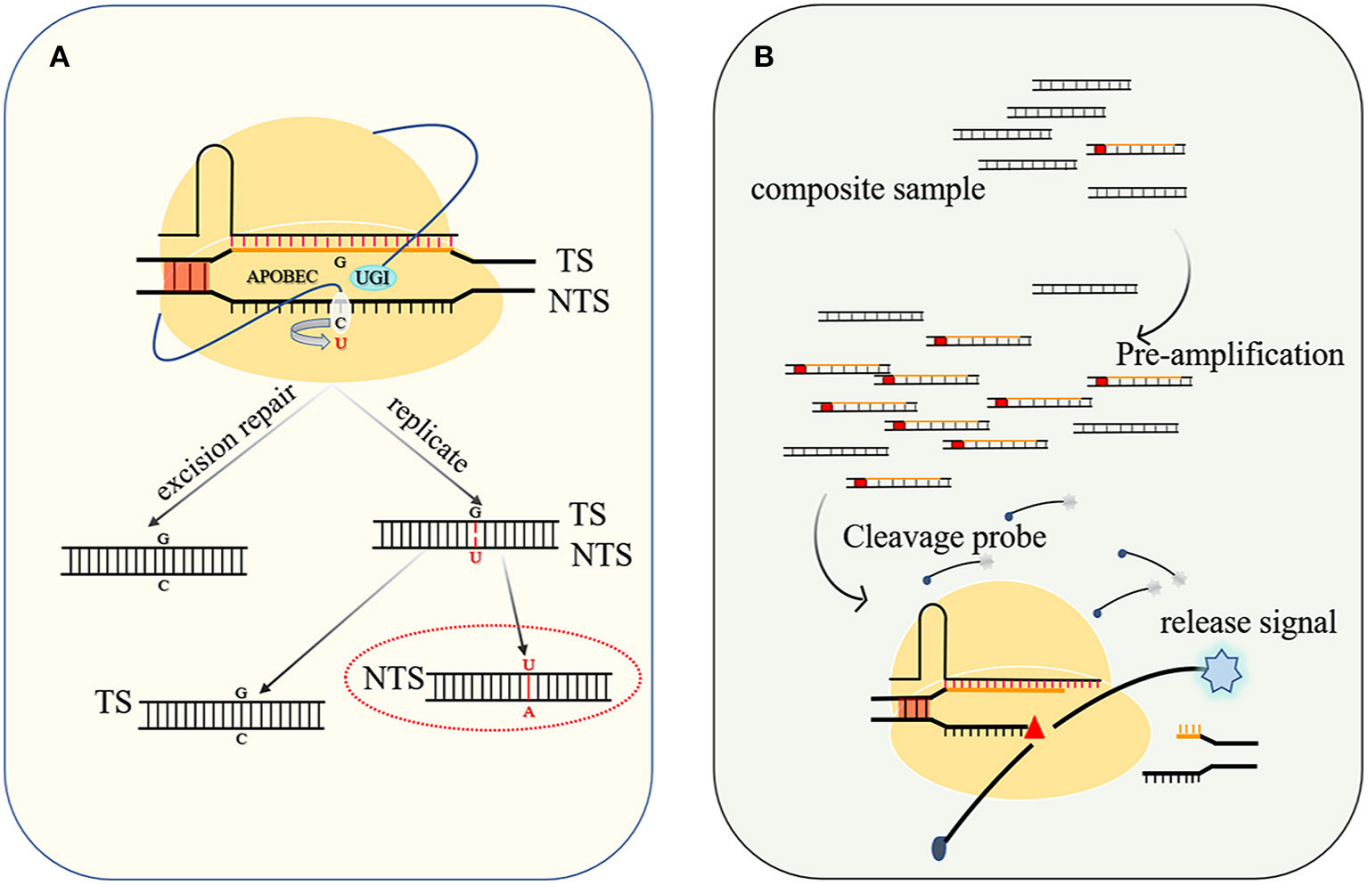
Base editing diagram and principle of nucleic acid detection. **(A)** When UGI is used to inhibit host base excision repair, APOBEC catalyzes the conversion of C to U with higher efficiency, and after DNA replication, a G–C base pair is changed to an A–U (T) base pair. **(B)** The targeted nucleic acid undergoes preamplification to increase its concentration and is cleaved by the Cas protein to activate the trans-cleavage activity of the Cas protein and decomposes the probe to release the detection signal.

The application of CRISPR technology has led to tremendous progress in genetic engineering, but the precise editing of single nucleotides is still a challenge. Aiming to achieve this goal, the rat cytosine deaminase APOBEC and uracil DNA glycosylase (UDG) inhibitor (UGI) were, respectively, fused to the N- and C-termini of dCas12a. After dCas12a binds to the target site, the R-loop is formed, and the non-complementary ssDNA strand provides the catalytic substrate for APOBEC. Consequently, the cytosine (C) at the 8–13 nt position downstream of the PAM was deaminated to uracil (U), and the previous C–G base pair was successfully changed to an A–T base pair after DNA replication. At the same time, the C-terminal UGI can inhibit base excision repair by UDG and increase the mutation rate (Figure 4A) (Banno et al., 2018). The same strategy was applied to dCas9, but researchers found that dCas12a-mediated BE had higher editing efficiency and lower off-target rate (Li X. et al., 2018). Although it can improve the efficiency of BE to replace dCas9 with nCas9, nickase activity of nCas9 leads to DNA damage response (DDR) (Wang et al., 2020a). The targeted chromosomal single-base mutations made feasible by this CRISPR-based base editor have great application value in protein engineering and gene therapy.

## Targeting ssDNA by The Type V Family

### Specific ssDNA Cleavage Activity

Many of the dsDNA-targeting CRISPR effector proteins belonging to class II have shown concurrent ssDNA-targeting cleavage *in vivo* and *in vitro* (Table 1) (Ma et al., 2015). This ssDNA cleavage activity is not limited by the PAM. As long as the target substrate is complementary to the guide sequence of the crRNA, it will work. The recently identified type V-F system almost only occurs in a group of archaea characterized by small cell and genome sizes, and the signature Cas14 protein of this system is also very compact, about 400–700 amino acids, which is half the size of the effector proteins in other type V systems. The mature tracrRNA and crRNA were obtained through environmental meta-transcriptomic sequence analysis. When these genes were expressed in a heterologous host, although Cas14 could successfully bind the gRNA to form a binary complex, no catalytic activity was observed with either single- or double-stranded DNA or RNA *in vivo*. However, when the purified Cas14–sgRNA binary complex was incubated with complementary ssDNA *in vitro*, cleavage activity was observed (Harrington et al., 2018; Savage, 2019). Although no *in vivo* Cas14 catalytic activity has been detected for the time being, it may be due to the lack of some key factors contained in the natural host of Cas14, which is worthy of further exploration. It also implies that targeted ssDNA cleavage is a widespread and possibly ancestral function.

### Non-specific ssDNA Cleavage Activity

Type V family proteins have unique non-specific ssDNA cleavage activity (Table 1). After the effector protein cleaves the targeted dsDNA substance, the PAM-distal product is released, but the Cas protein is still bound to the PAM-proximal strand. At this point, the RuvC domain can still access ssDNA substrates and perform non-specific cleavage, which is also known as the promiscuous cleavage of collateral ssDNA or trans-cleavage (Li et al., 2018a; Liu J. J. et al., 2019; Swarts and Jinek, 2019).

### Applications Based on ssDNA Cleavage Activity

#### Nucleic Acid Detection Platform

The trans-acting DNase activity induced by DNA-specific recognition has been developed into a DNA detection platform by employing a quenched fluorescent ssDNA as the probe. When a cognate target is present in the system, Cas12a severs the targeted entities to activate trans-cleavage activity, thereby also cleaving the surrounding probes to release a detectable fluorescent molecule (Figure 4B). Preamplification of the target sequence by isothermal recombinase-polymerase amplification (RPA) or loop-mediated isothermal amplification (LAMP) can improve the sensitivity, and the detection limit of the desired DNA or RNA can be as low as 10 aM (attomolar) (Li et al., 2018b). After continuously optimizing this detection system and improving the specificity of recognition, the system has been widely used in the discrimination of single nucleotide polymorphisms (Teng et al., 2019a) and genotyping (Harrington et al., 2018) and virus detection, such as severe acute respiratory syndrome coronavirus 2 (SARS-CoV-2) (Broughton et al., 2020; Wang et al., 2020b).

#### Small Molecule Detection Platform

Liang et al. combined the sensitivity of allosteric transcription factors (aTFs) for small molecules with the non-specific ssDNA cleavage activity of Cas12a to develop a platform for detecting small molecules, named CaT-SMelor (CRISPR-Cas12a and aTF-mediated small molecule detector). It is well-known that aTFs have an effector-binding domain and a DNA-binding domain. Following preassembly of the well-designed dsDNA that can be recognized by Cas12a with aTF, a targeted small molecule can bind to the aTF, which then releases the dsDNA. Cas12a in turn binds to the released dsDNA and activates the trans-activity to cut the nearby quenched fluorescently labeled ssDNA and to generate a fluorescent detection signal. This system can successfully differentiate nanomolar levels of target molecules from a structurally similar analog (Liang et al., 2019).

## Targeting ssRNA by The Type V Family

Type V-G is a recently identified CRISPR system with a small signature protein, Cas12g, which has an intact RuvC-like endonuclease domain at the C-terminus (Yan et al., 2019). However, no ssDNA or dsDNA cleavage activity was detected either *in vivo* or *in vitro* when Cas12g was incubated with crRNA and tracrRNA. Surprisingly, when the substrates were replaced with cognate ssRNA, Cas12 exhibited targeted cleavage activity without constraint of a PAM sequence. Moreover, after binding to the targeted RNA, the trans-cleavage activity was activated to non-specifically cleave the surrounding ssRNA. Inactivation of the RuvC domain determined that Cas12g's RNA cleavage activity depends on an intact RuvC domain, disproving the previous theory that the RuvC domain could only act on DNA substrates. Although the effector protein Cas13 of the type VI CRISPR system also has RNA-targeting cleavage activity, it cuts RNA *via* two HEPN domains (Liu et al., 2017).

### Applications Based on ssRNA Recognition

Cas12g has similar functions as Cas13, including RNA-guided RNA-targeting and trans-cleavage activity. With the further study of Cas13, a variety of RNA-specific tools have been developed. Cas12g also has the full corresponding development potential, and its size is only 700 aa. Moreover, its origin from a hot spring metagenome gives it high thermal stability.

#### RNA-Based Nucleic Acid Detection Platform

Gootenberg et al. developed a nucleic acid detection platform based on the type VI CRISPR enzyme Cas13. The system is based on preamplifying DNA or RNA samples through RPA or RT-RPA, T7 RNA polymerase transcription of amplified DNA to RNA, and detection by Cas13 *via* collateral cleavage of an ssRNA probe (Gootenberg et al., 2017). Although Cas13 and Cas12a can only use RNA and DNA as recognition matrices, respectively, because of the amplification steps and the flexible use of transcription and reverse transcription, these two detection platforms can detect samples whose initial substrates are either DNA or RNA. Therefore, when the sample cannot be amplified and transformed or the detection purpose does not allow the preamplification step, the detection of DNA or RNA requires the division of the two platforms. For example, Chen et al. used a nucleic acid detection platform to monitor the degree of RNA methylation. Because methylated nucleotides are not complementary to crRNA, the intensity of the fluorescent signal reflects the methylation degree of RNA (Chen Y. et al., 2019). For this purpose, the Cas13-based RNA nucleic acid detection platform is the only option available to date. If Cas12g instead of Cas13 is developed as a portable *in vitro* RNA detection kit, thermal stability is a potential advantage (Chen et al., 2020).

#### RNA Single-Base Editing

Adenosine deaminase acting on RNA type 2 (ADAR2) has an N-terminal dsRNA-binding domain and a C-terminal catalytic deamination domain, which can be combined with dsRNA to achieve adenosine-to-inosine transformation through hydrolytic deamination. Furthermore, the ADAR2 deaminase domain (ASAR2_DD_) can function independently without other cofactors. Cox et al. tethered the hyperactivating mutant ADAR2_DD_ to the catalytically inactive Cas13b (dCas13b) and designed the gRNA to introduce A:C mismatches at the desired site (ADAR2 characteristics, higher catalytic activity for A:C mismatch sites on dsRNA) to achieve precise editing of A to I in mammals (Cox et al., 2017). Through multiple rounds of evolution of ADAR2_DD_, the team obtained a cytosine deaminase with the ability to convert cytosine to uracil and fused this evolved cytidine deaminase to dCas13 to develop a programmable RNA editing tool for specific C to U exchange (Abudayyeh et al., 2019; Aquino-Jarquin, 2020). Cas13b has been shown to be free of PFS (protospacer-flanking site, parallel to PAM in class II) restrictions in mammalian cells, compared with DNA base editors where the target site is restricted to PAM. The RNA base editor based on Cas13 is more flexible and expands the target range in gene therapy, but still faces challenges in viral delivery. Not only does Cas12g have no PAM restrictions, but minimizing the system by using Cas12g is a potential optimization opportunity.

## Conclusion and Outlook

Among the diverse CRISPR–Cas systems, class 2 has the most extensive applications because of having only one effector protein. The type V CRISPR–Cas system has become a shining star due to the growing type V family and the diversity of its functions. Nevertheless, only the earliest Cas12a and Cas12b have been used in practical applications. In this review, we summarize all the type V family systems discovered so far and discuss their characteristics in a taxonomic manner. Some of these newly discovered type V family Cas proteins can be used as candidates in the novel application strategies that are currently being developed. Some of their unique properties such as high mismatch sensitivity, small size, and thermal stability have the potential to further optimize these tools.

Except for Cas12a, Cas12b, and Cas12e, the N-terminal domain and crystal structures of other type V family Cas proteins have not been resolved. Through the comparison of Cas12a, Cas12b, and Cas12e, it is not difficult to realize that the diversity of the N-terminal domain of the Cas protein reflects the differences in the mechanism of action and the cleavage effect. Future analysis of the structure of these Cas proteins will further reveal the relationship between structure and function, which will contribute to the further development and optimization of the CRISPR–Cas system.

In addition to type V-U5, the functions of several other subtypes of the type V-U family (types V-U1, V-U2, V-U3, V-U4) are unknown. Behind the unusual may be born with surprise. In addition, the effector proteins in these families are very similar to TnpB, so further research on the type V-U family will help explain how CRISPR–Cas effectors evolved from TnpB.

## Author Contributions

BT wrote the manuscript and performed all of the necessary literature searches. HD, YC, and PJ performed the necessary literature searches. ZJ revised the manuscript and gave valuable suggestions. DZ designed the review, reviewed it, and approved the submitted manuscript.

## Conflict of Interest

The authors declare that the research was conducted in the absence of any commercial or financial relationships that could be construed as a potential conflict of interest.
